# Supplementary material for: TADA—a machine learning tool for functional annotation-based prioritisation of pathogenic CNVs
Source: Genome Biol. 2022 Mar 1;23:67. doi: 10.1186/s13059-022-02631-z (PMC8886976; doi:10.1186/s13059-022-02631-z)
Supplement: Supplementary file 1 — Additional file 1 Figures S1-S21. Tables S1-3. [file 13059_2022_2631_MOESM1_ESM.pdf]

Supplementary Material

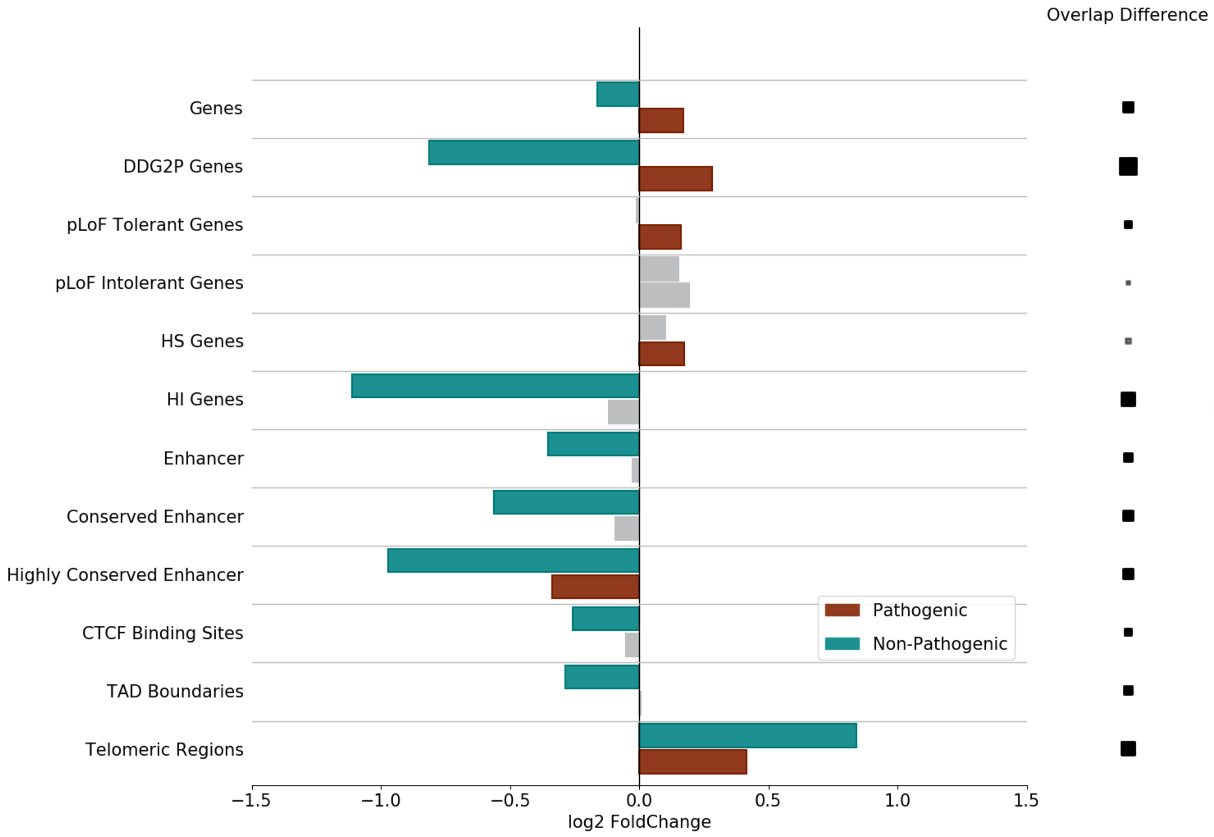

Figure S1. Enrichment Analysis of non-pathogenic and pathogenic duplications. Non-pathogenic duplications are significantly depleted in coding and regulatory regions as well as TAD boundaries. In contrast, pathogenic duplications are significantly enriched coding regions. Both non-pathogenic and pathogenic duplications are enriched in extended telomeric regions. Grey bars and squares indicate a non-significant FC ($q - value \leq 0.01$).

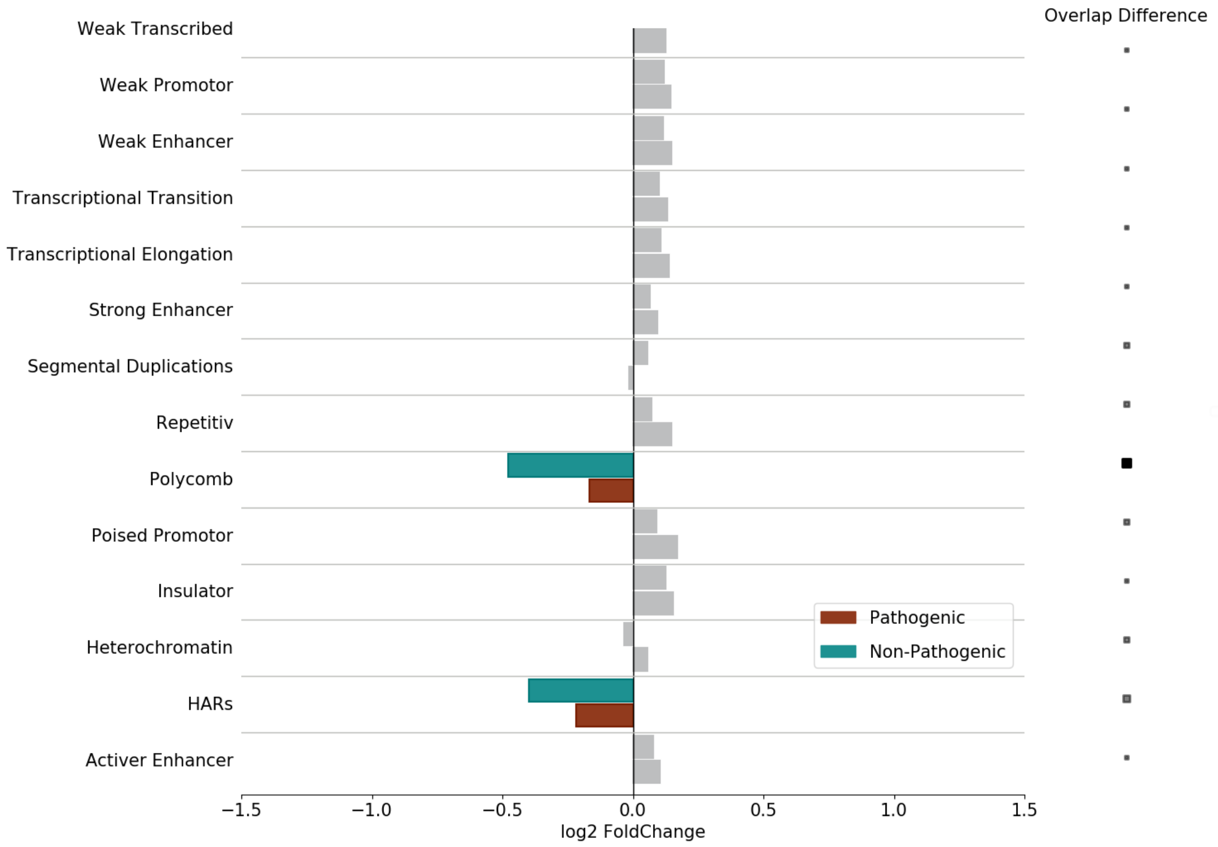

Figure S2. Enrichment of non-pathogenic and pathogenic deletions in ChromHMM annotation, HARs and Segmental Duplications. Pathogenic and non-pathogenic deletions are significantly depleted in polycomb-repressed and human-accelerated regions. Grey bars and squares indicate a non-significant FC ($q - value \leq 0.01$).

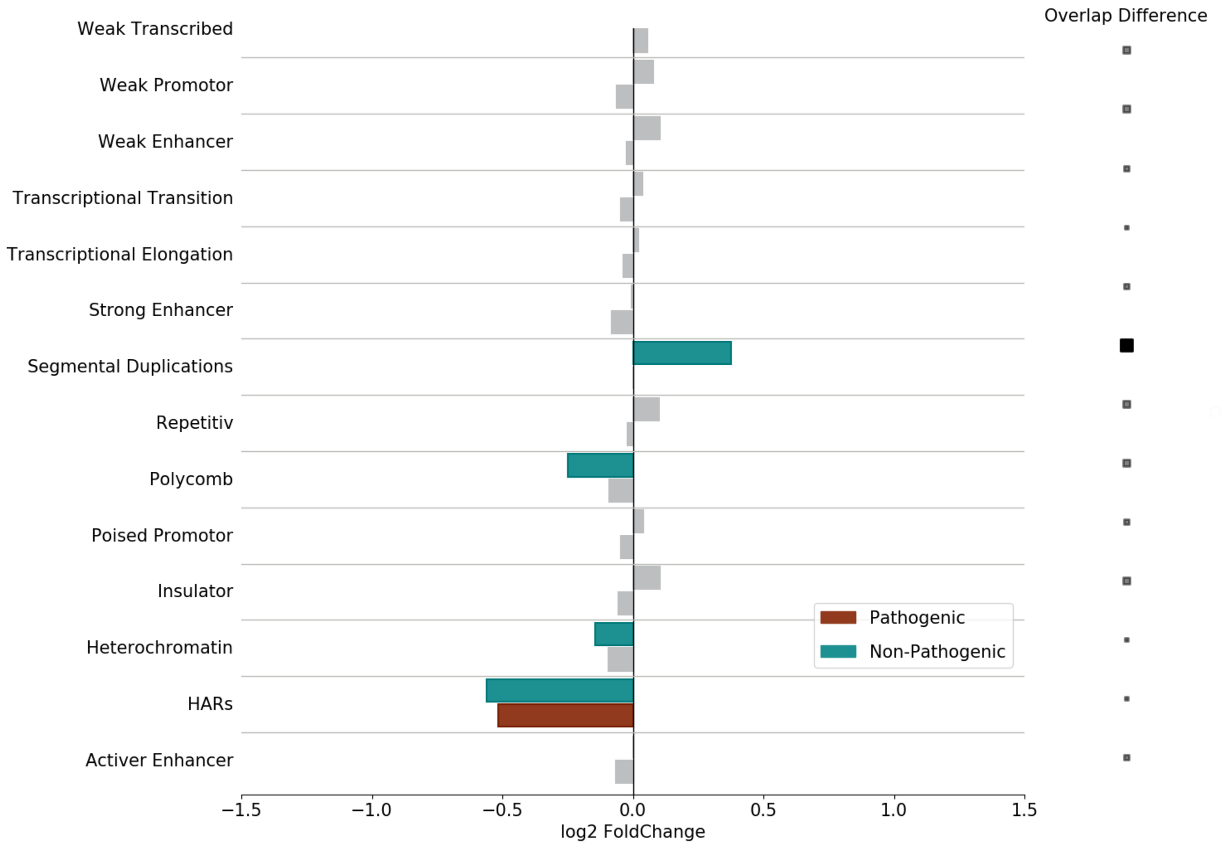

Figure S3. Enrichment of non-pathogenic and pathogenic duplications in ChromHMM annotation, HARs and Segmental Duplications. Non-pathogenic duplications are significantly enriched segmental duplications and significantly depleted in polycomb-repressed, heterochromatin and human-accelerated regions. Pathogenic duplications are significantly deplete in HARs. Grey bars and squares indicate a non-significant FC ($q - value \leq 0.01$).

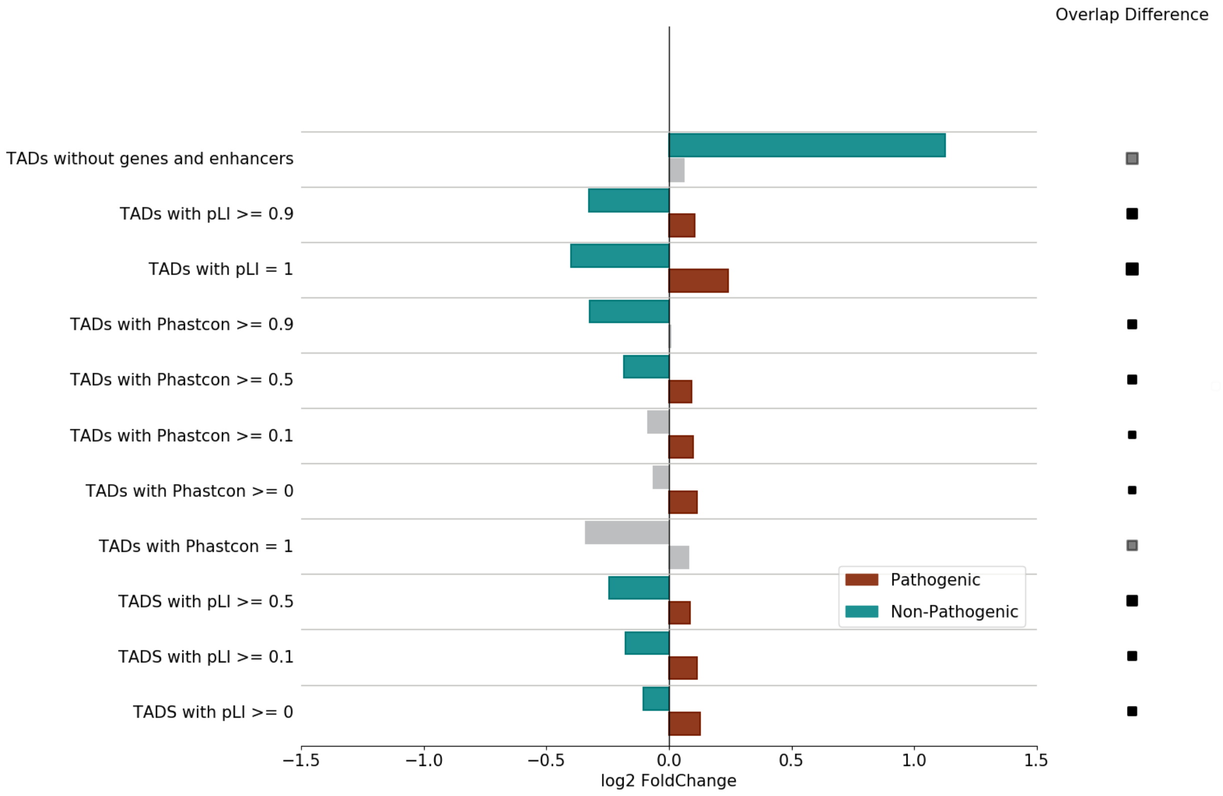

Figure S4. Enrichment of non-pathogenic and pathogenic deletions in TAD-centric annotation. We observe strong significantly enrichment of non-pathogenic deletions in TADs without gene or enhancer annotations but significant depletion in almost all TADs containing coding or regulatory annotation. Pathogenic deletions tend to be enriched in TADs with gene or enhancer annotation. Grey bars and squares indicate a non-significant FC ($q - value \leq 0.01$).

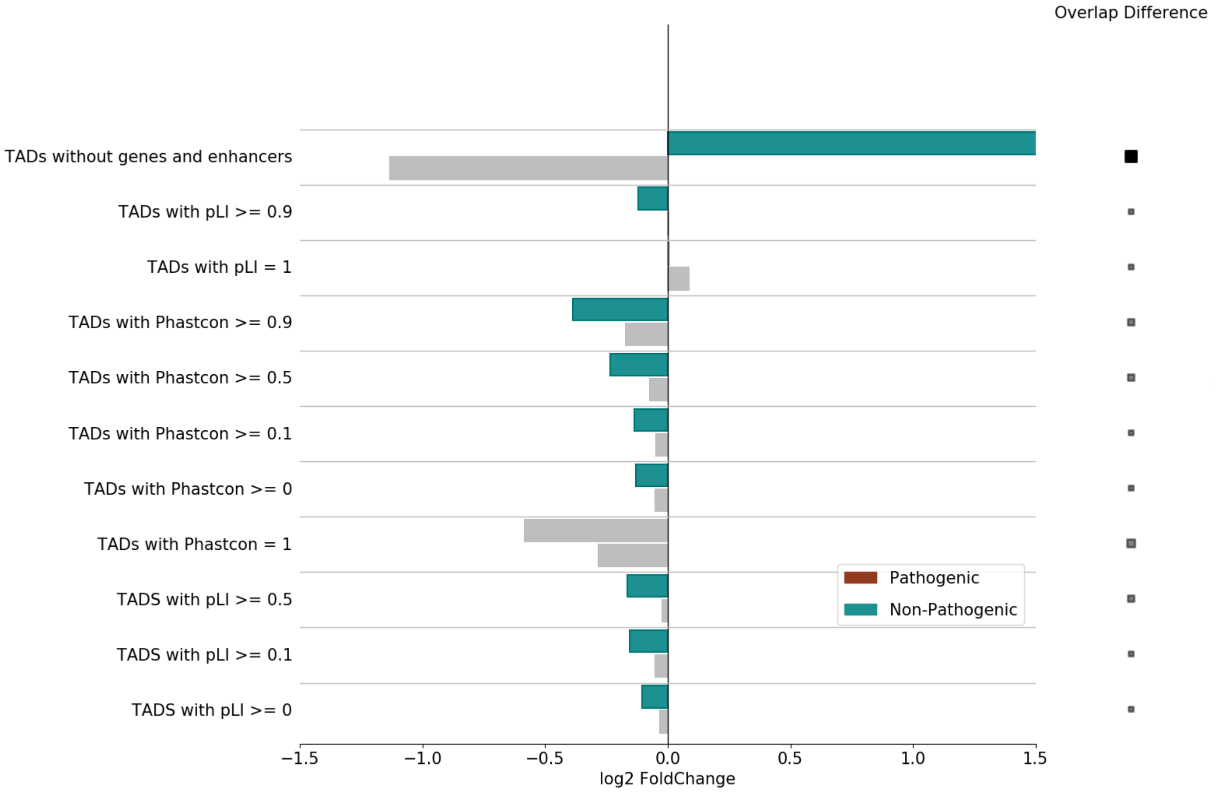

Figure S5. Enrichment of non-pathogenic and pathogenic duplications in TAD-centric. While we are not able to observe any significant enrichment or depletion of pathogenic duplications in TAD-centric annotation, we observe significant enrichment of non-pathogenic duplications in TADs without gene or enhancer annotation. Non-pathogenic duplications are also significantly depleted in most TADs with coding or regulatory annotations. Grey bars and squares indicate a non-significant FC ($q - value \leq 0.01$).

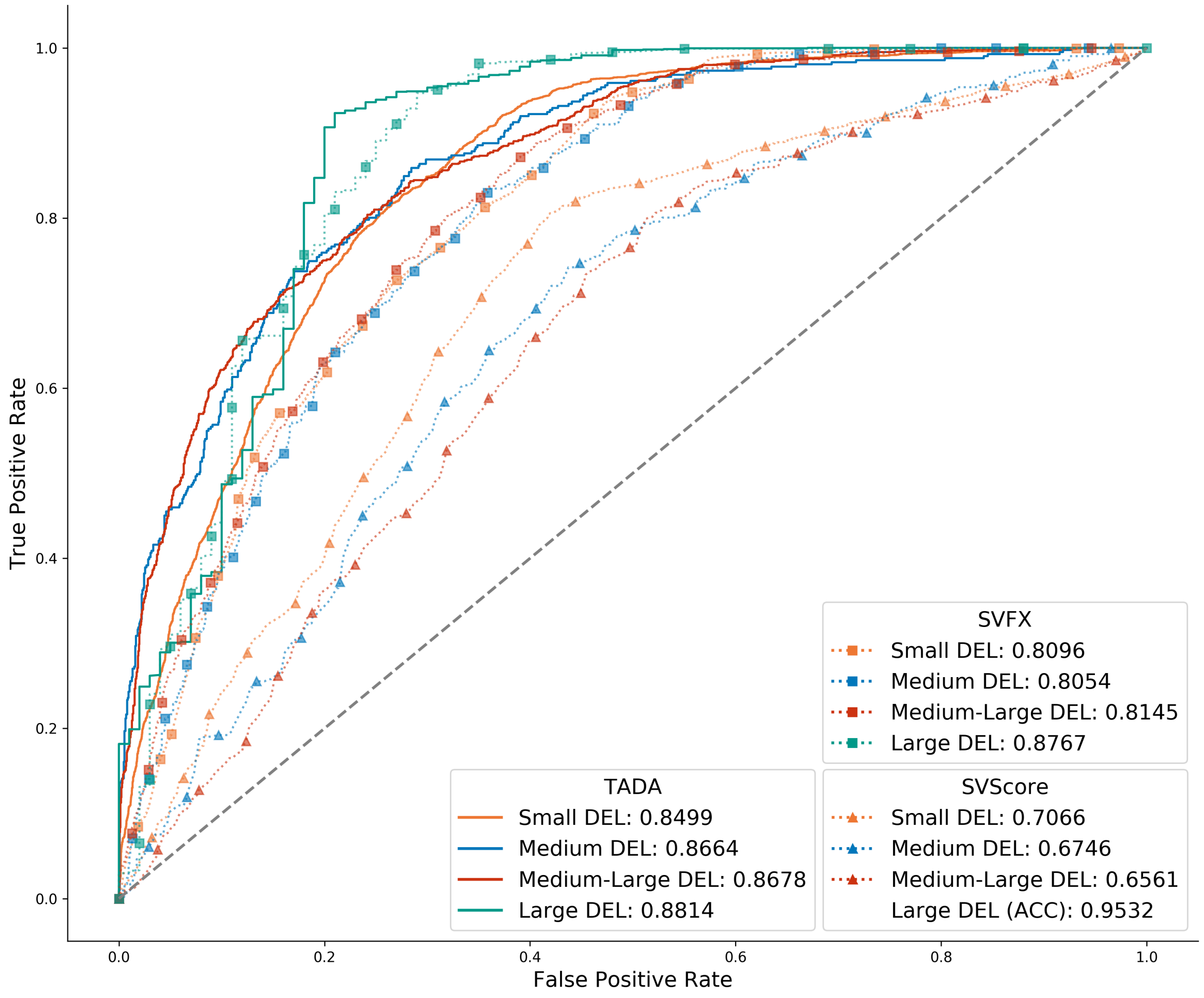

Figure S6. ROC-Curves showing the classification performance of TADA, SVScore and SVFX on ClinVar variants separated into non-overlapping groups by size: Small (< 50kb), Medium (< 100kb), Medium-Large (< 1mb), Large (>= 1mb).

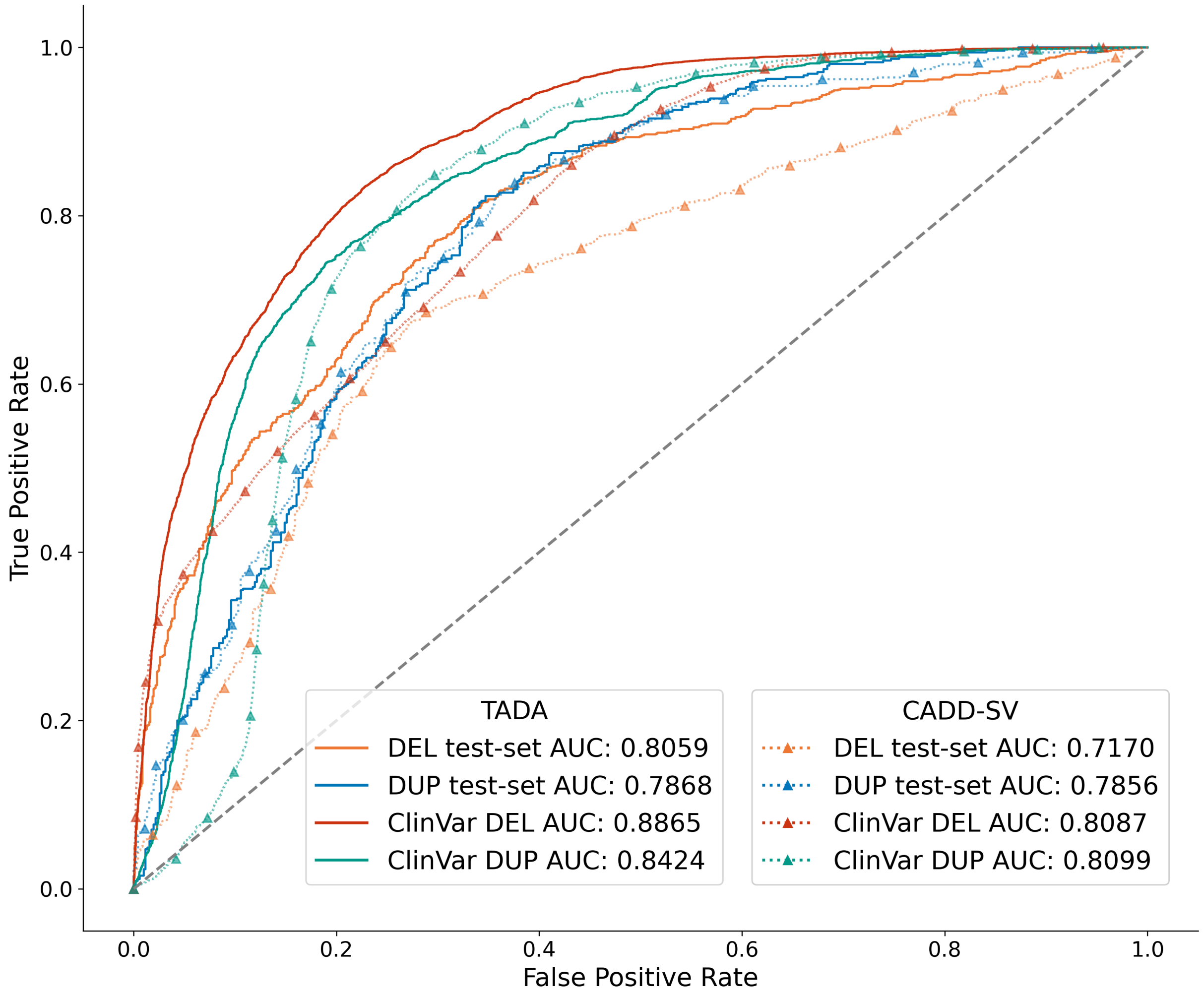

Figure S7. ROC-Curves showing the classification performance of TADA and CADD-SV on ClinVar and Test-Split CNVs.

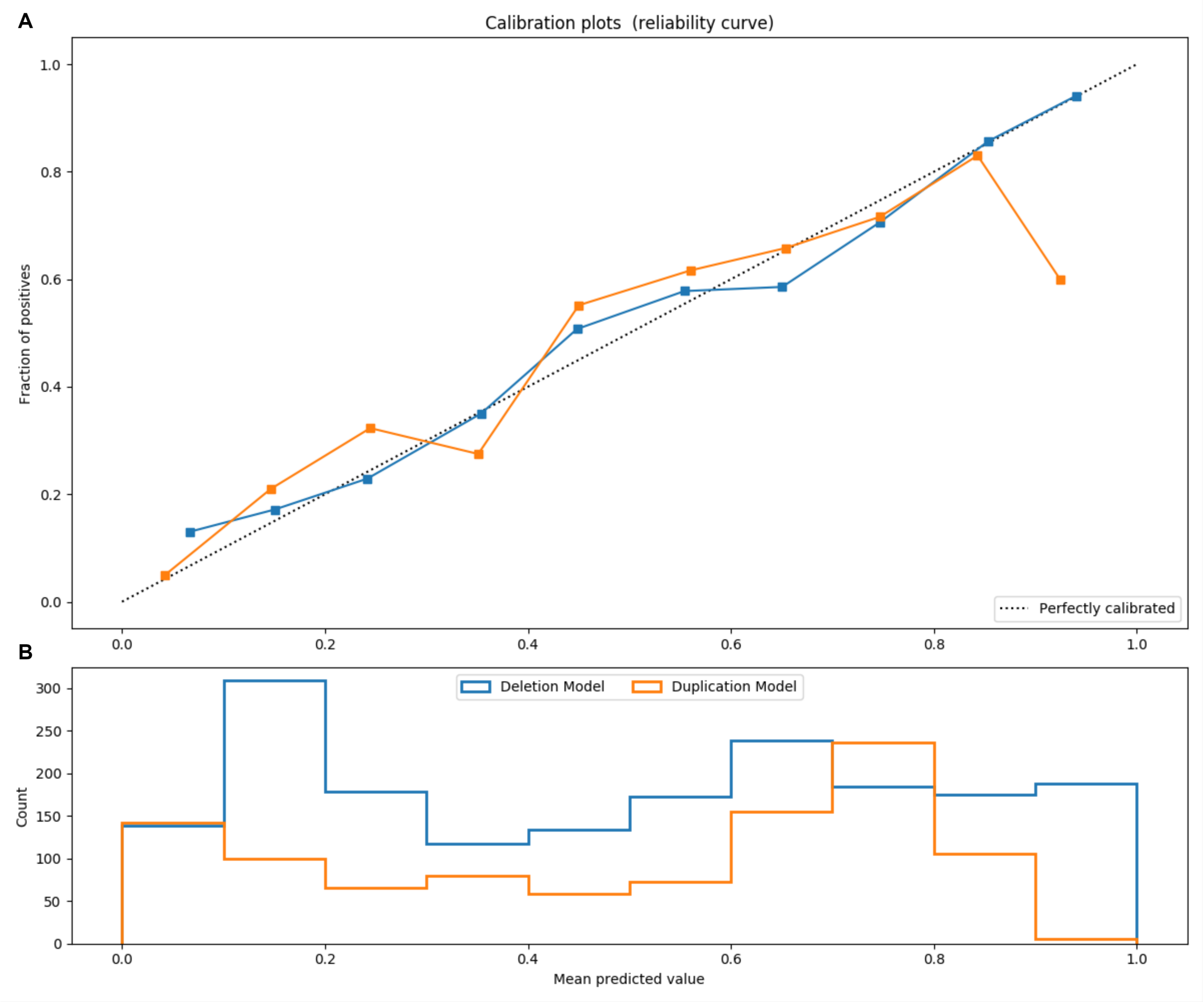

Figure S8. Calibration of the Predicted Class Probabilities for the Deletion and Duplication Model. **A** shows the fraction of positives vs the mean predicted value. **B** shows the absolute count of variants predicted over mean predictive values. The dotted line in the upper plot indicates perfect calibration.

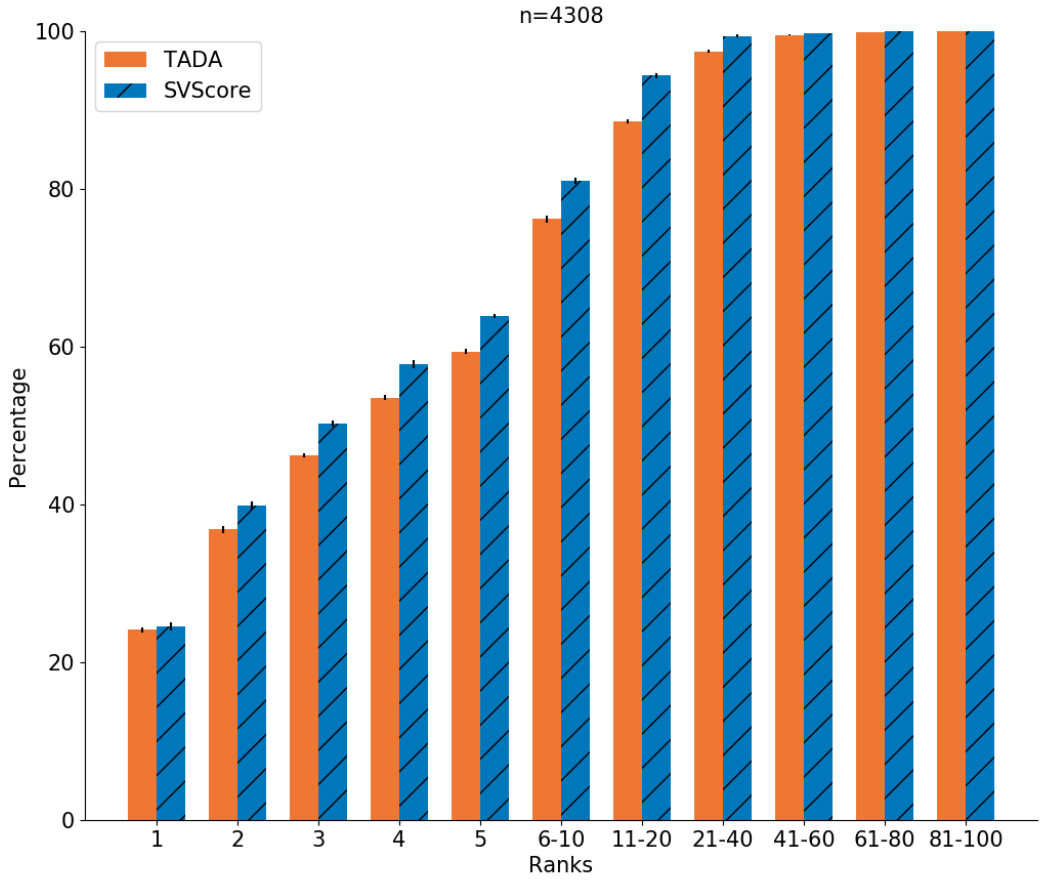

Figure S9. Ranking performance of TADA and SVScore for pathogenic ClinVar and rare GnomAD deletions.

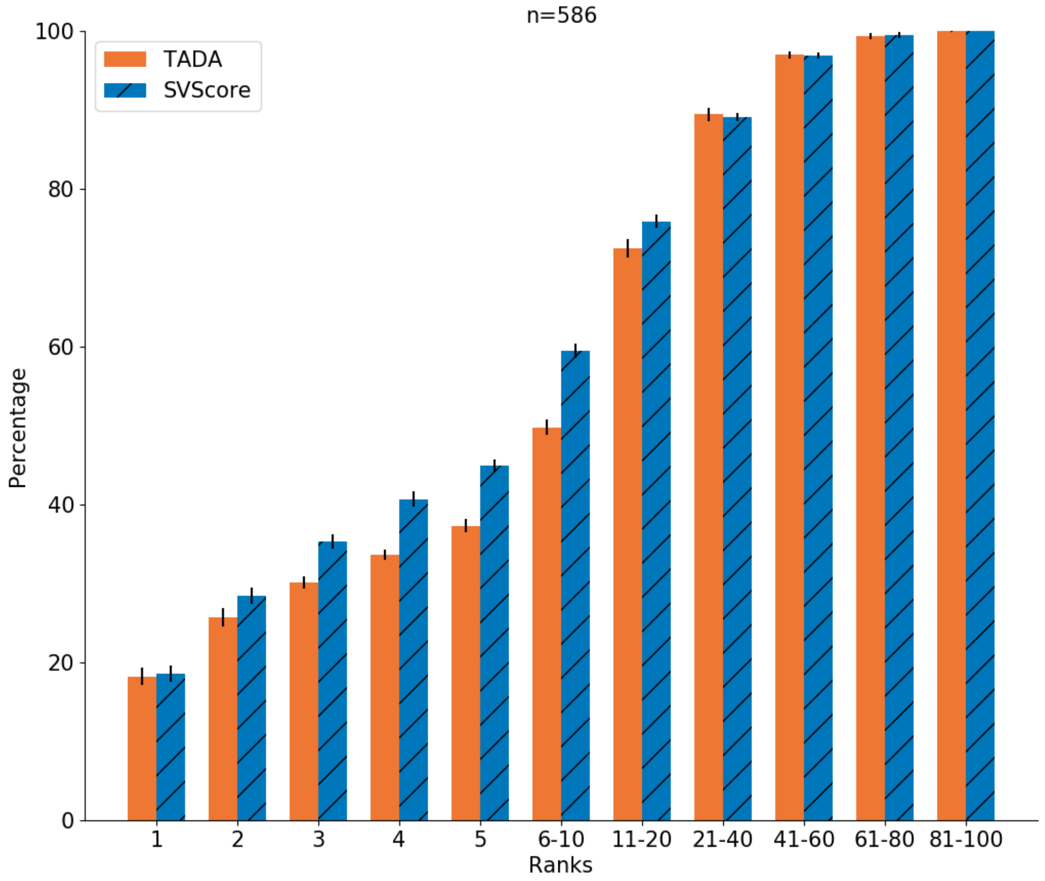

Figure S10. Ranking performance of TADA and SVScore for pathogenic ClinVar and rare GnomAD duplications.

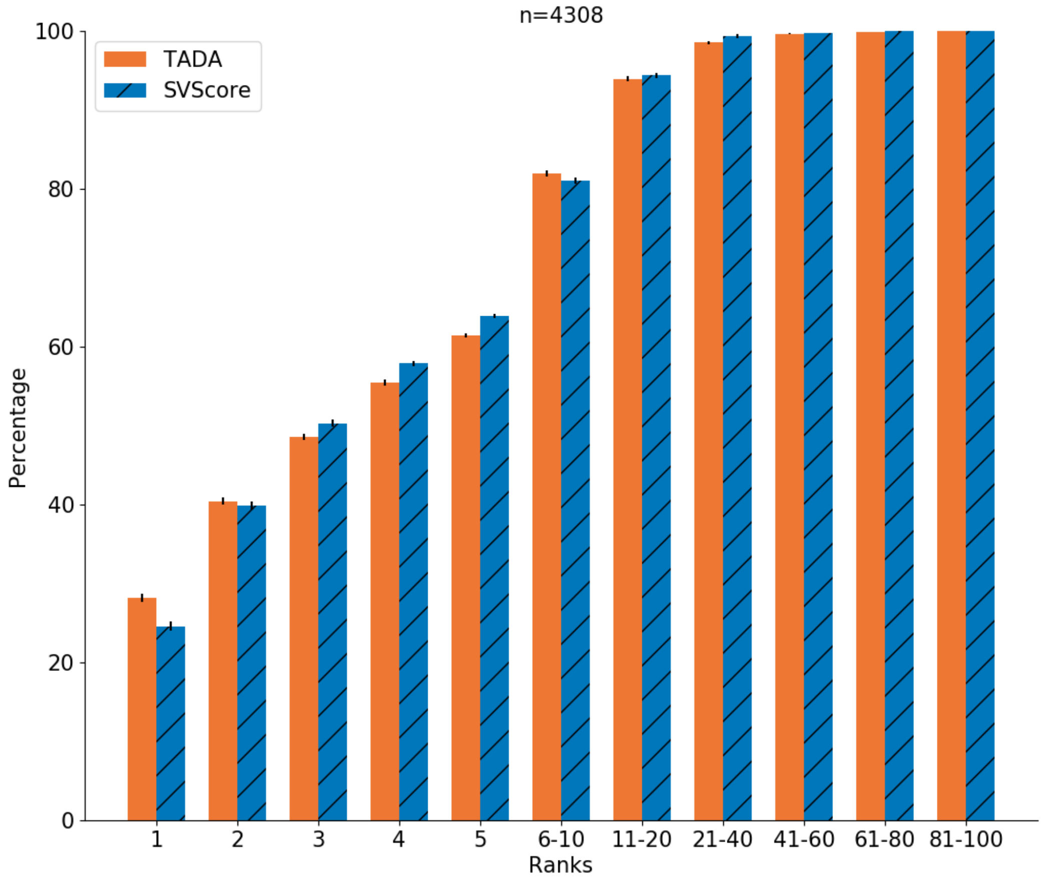

Figure S11. Ranking performance of the modified TADA classifier and SVScore for pathogenic ClinVar and rare GnomAD deletions.

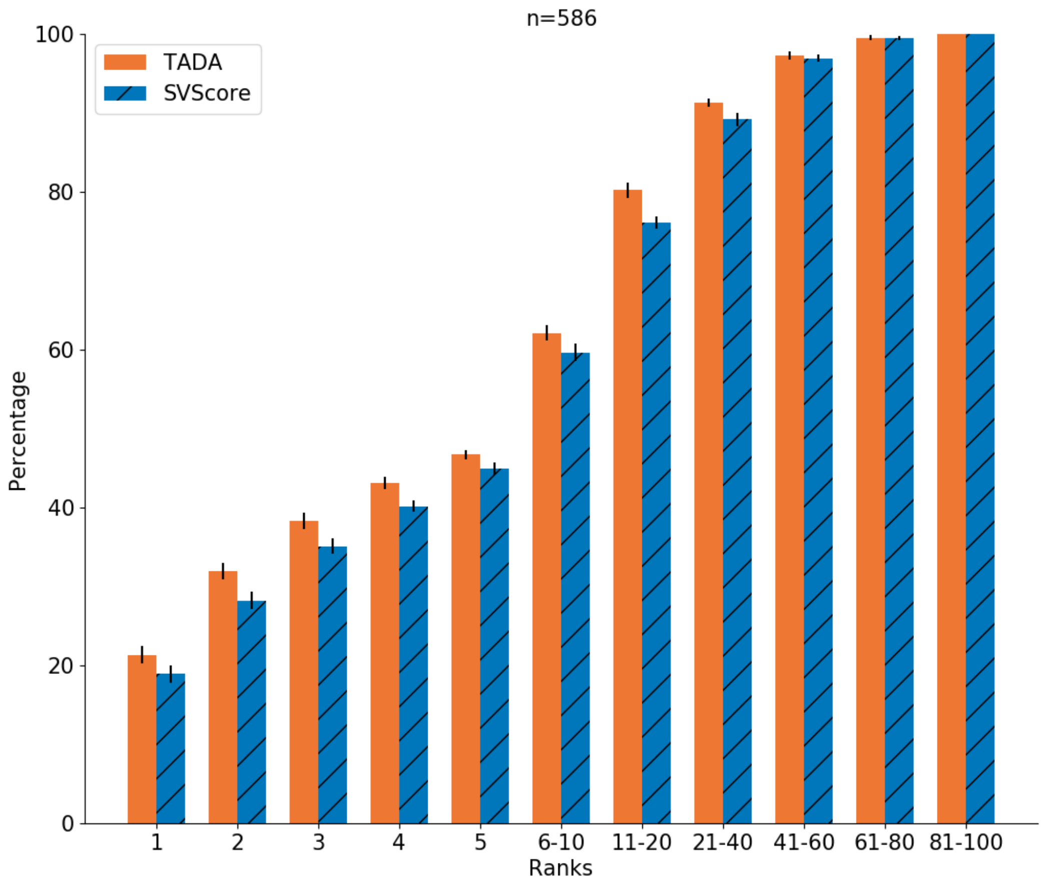

Figure S12. Ranking performance of the modified TADA classifier and SVScore for pathogenic ClinVar and rare GnomAD duplications.

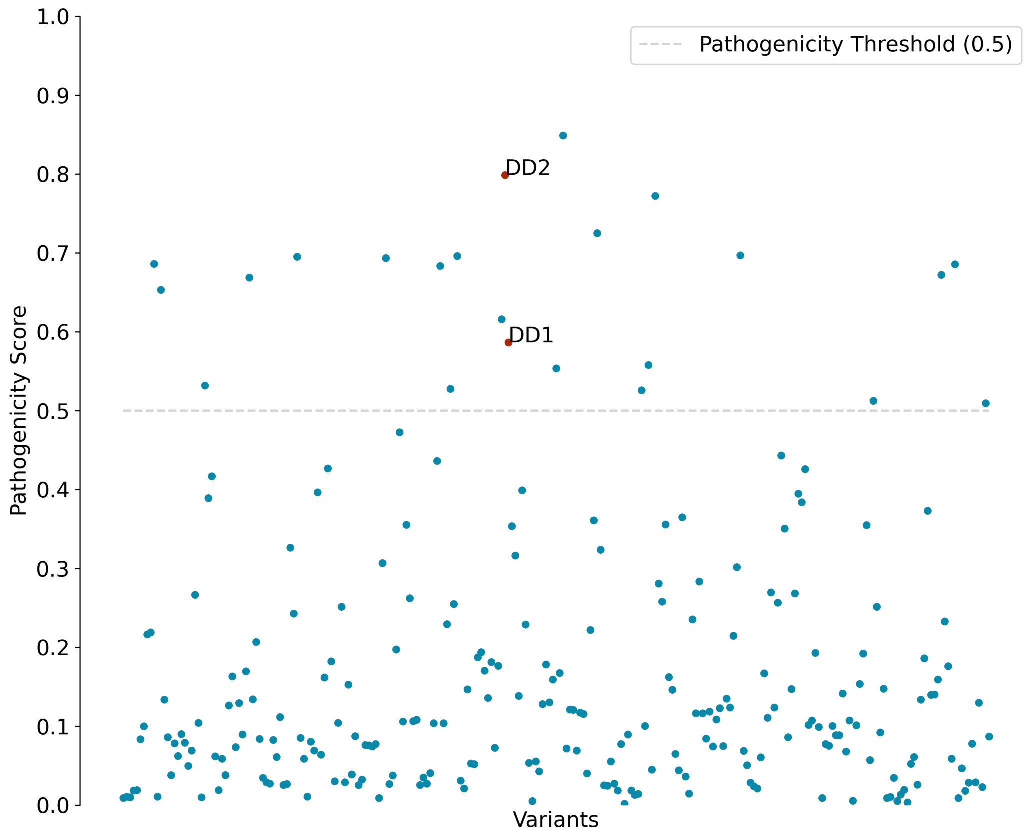

Figure S13. Pathogenicity Scores for Duplications of DD-Patients. The figure shows the computed pathogenicity scores for duplications of the DD2 patient including the pathogenic variant of DD1. Pathogenic duplications are marked in red. A potential threshold of 0.5 to separate duplications into pathogenic and non-pathogenic is indicated by the dashed line.

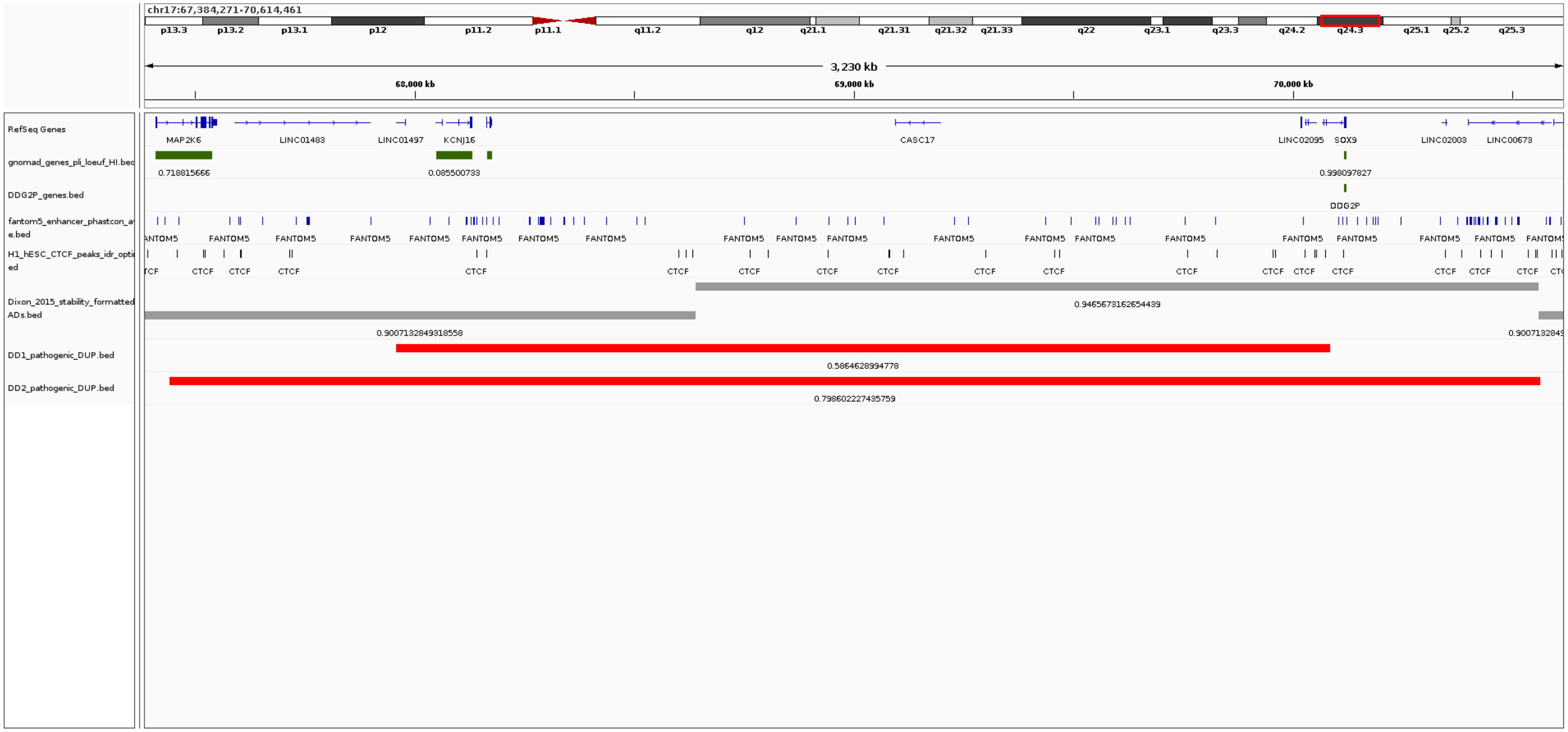

Figure S14. Regulatory Environment of DD-Patient Duplications. In this screenshot annotations of TADA are shown in combination with the disease-causing mutations of the DD1 and DD2 individual. The scores below the individual duplication is the computed pathogenicity score. The Genes in the GnomAD trakc also include their haploinsufficiency score.

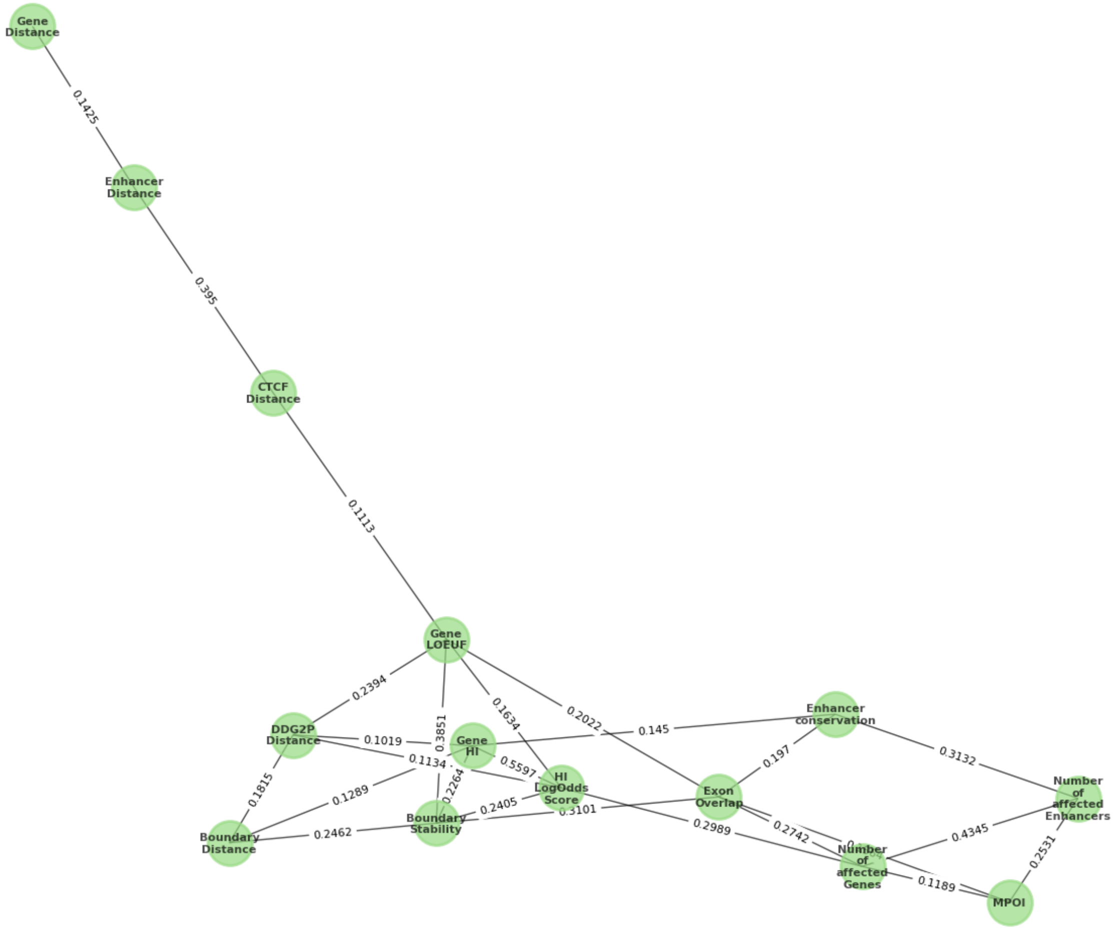

Figure S15. Partial Correlations between Features of the Deletion Model.

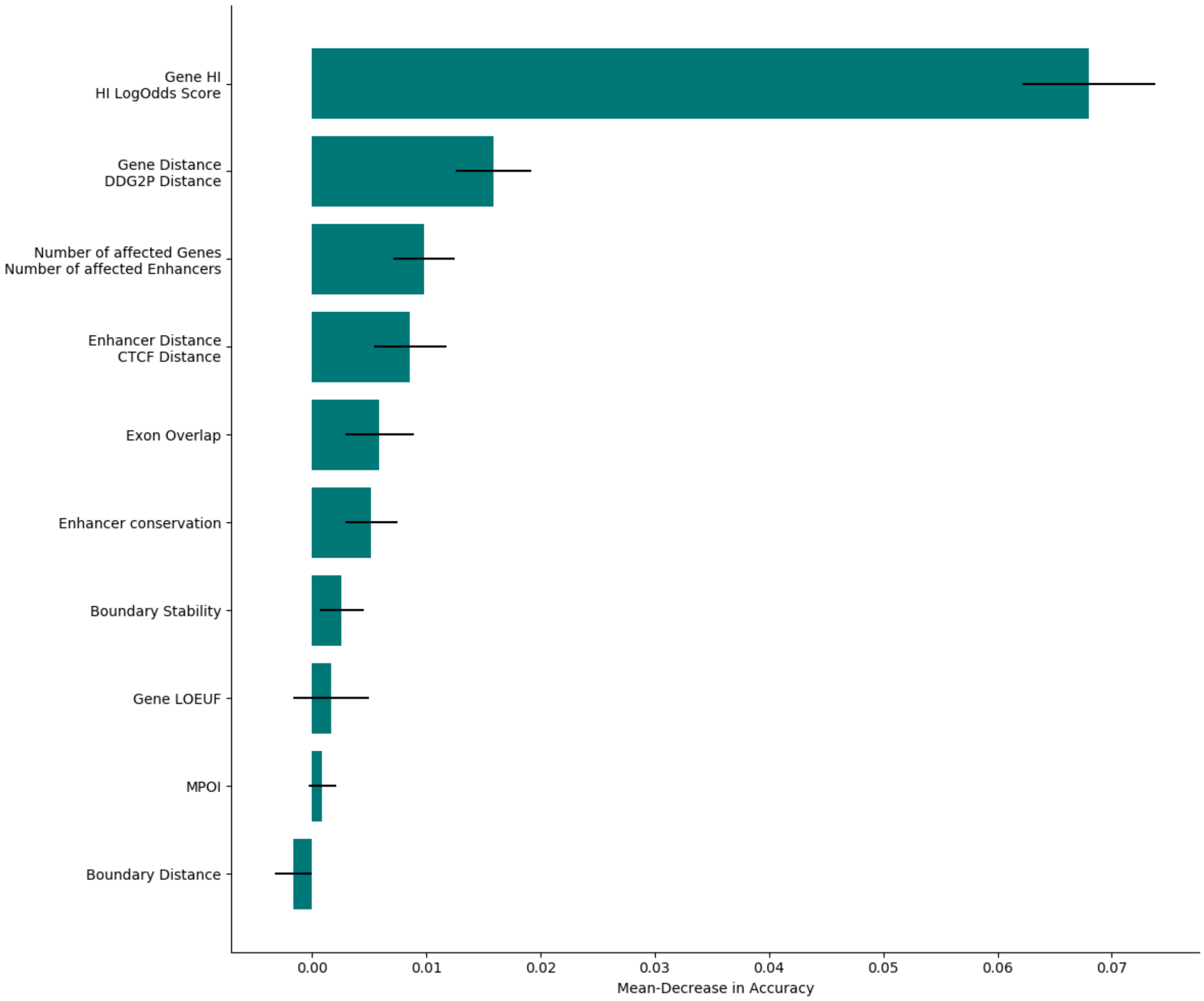

Figure S16. Feature Importance of the Duplication Model.

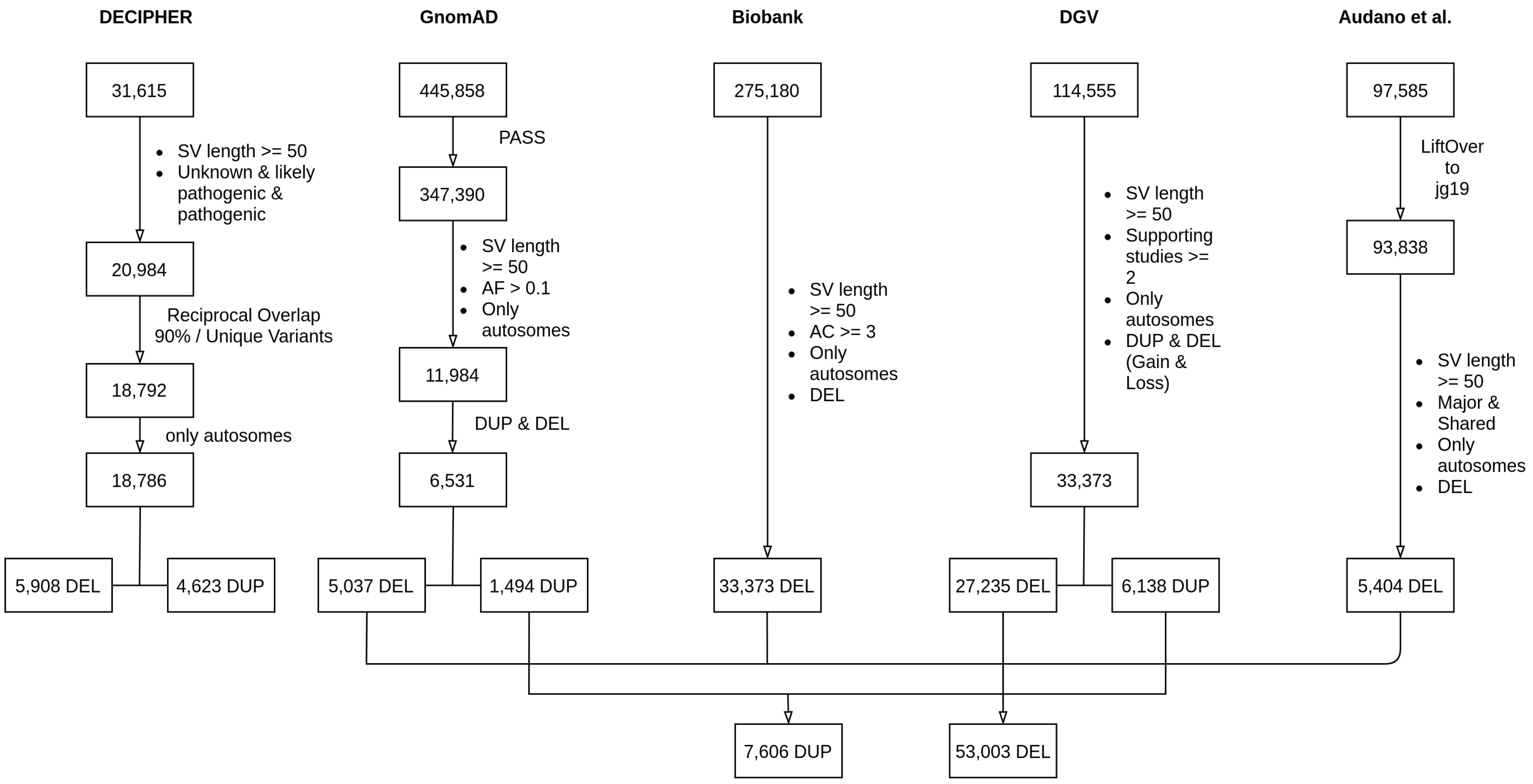

Figure S17. Number of Variants Pre- and Post-Filtering.

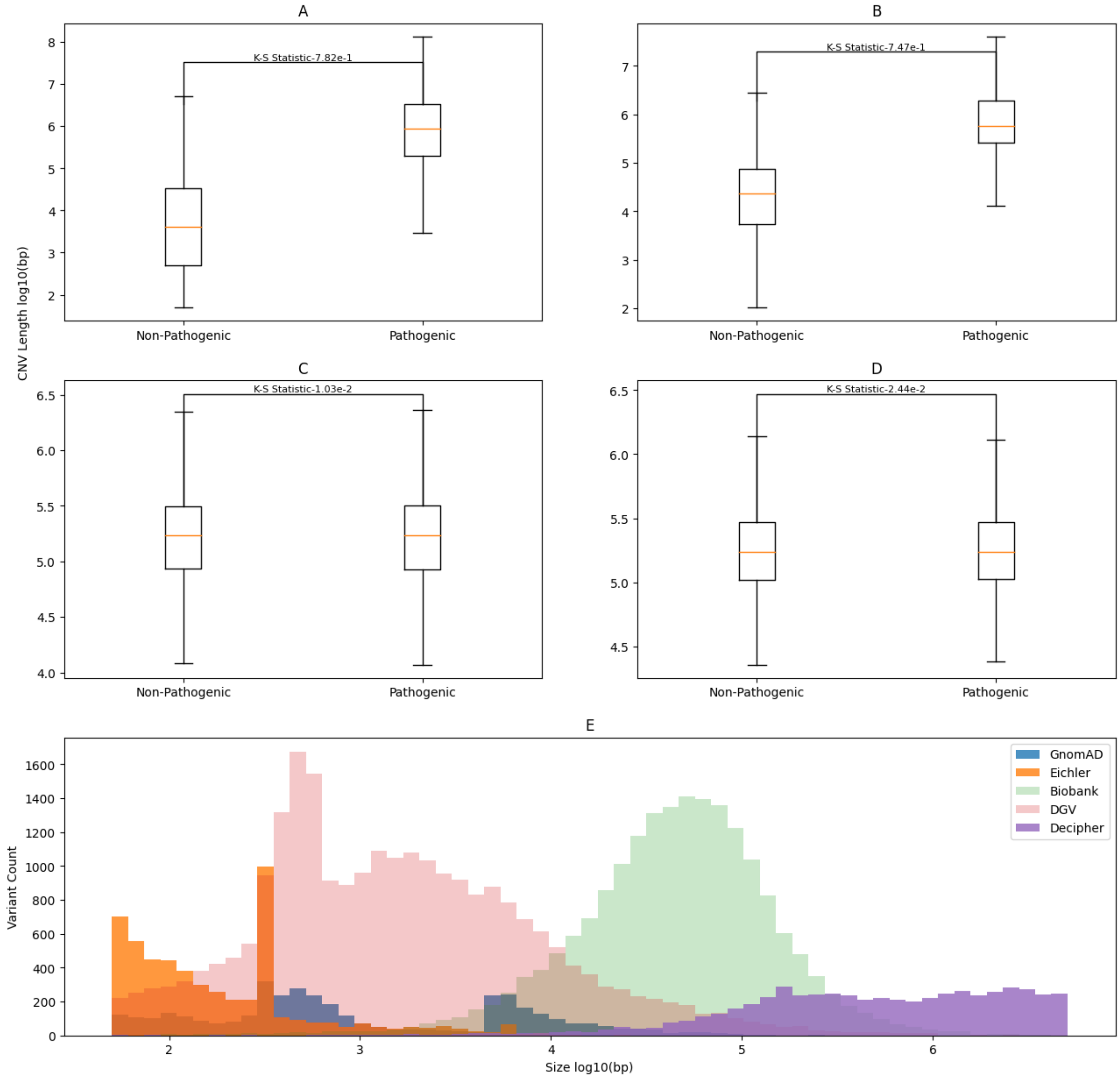

Figure S18. Size distribution of Pathogenic and Non-Pathogenic Variants. **A** and **C** show the comparison of size distributions between pathogenic and non-pathogenic variants before and after size-matching, respectively. **B** and **D** show the same comparison for duplications. **E** shows the size distributions of non-pathogenic and pathogenic deletions for each data source before size-matching.

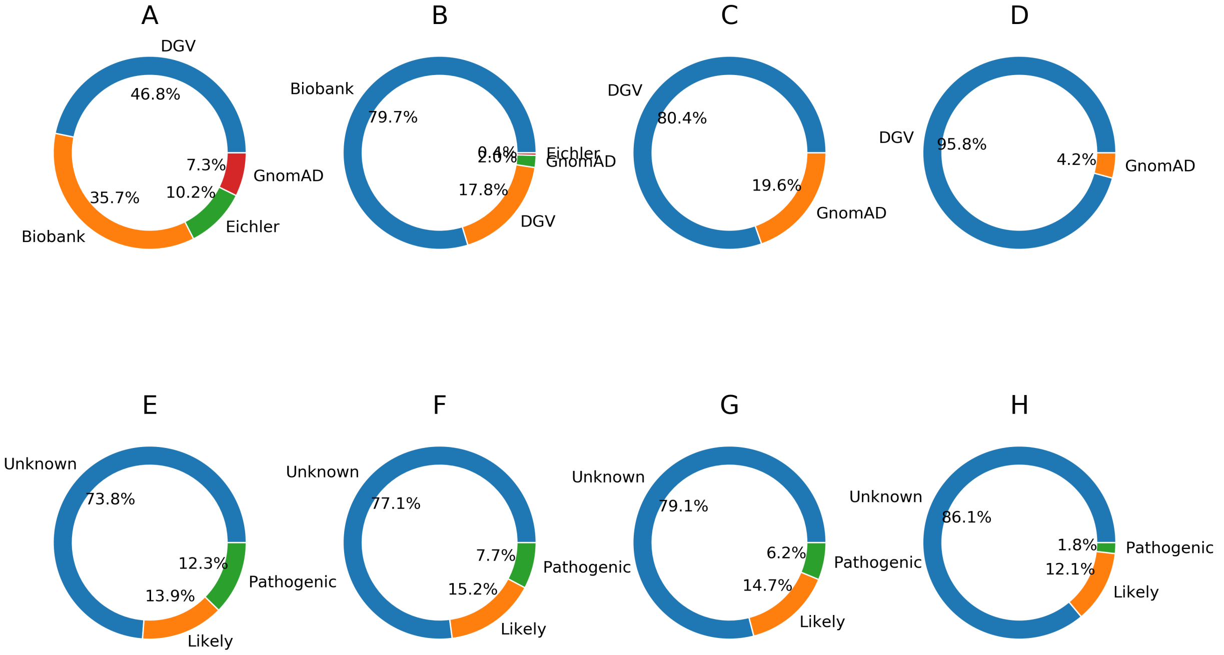

Figure S19. Proportion of Variants by Data Source and Pathogenicity. **A** and **B** show the proportion of non-pathogenic deletions by data source before and after size-matching, respectively. **C** and **D** show the same comparison for non-pathogenic duplications. **E**-**H** show the proportion of DECIPHER deletions and duplications by their annotated effect before and after size-matching, respectively. **I** shows the distribution of non-pathogenic variants by data source across the genome before size-matching.

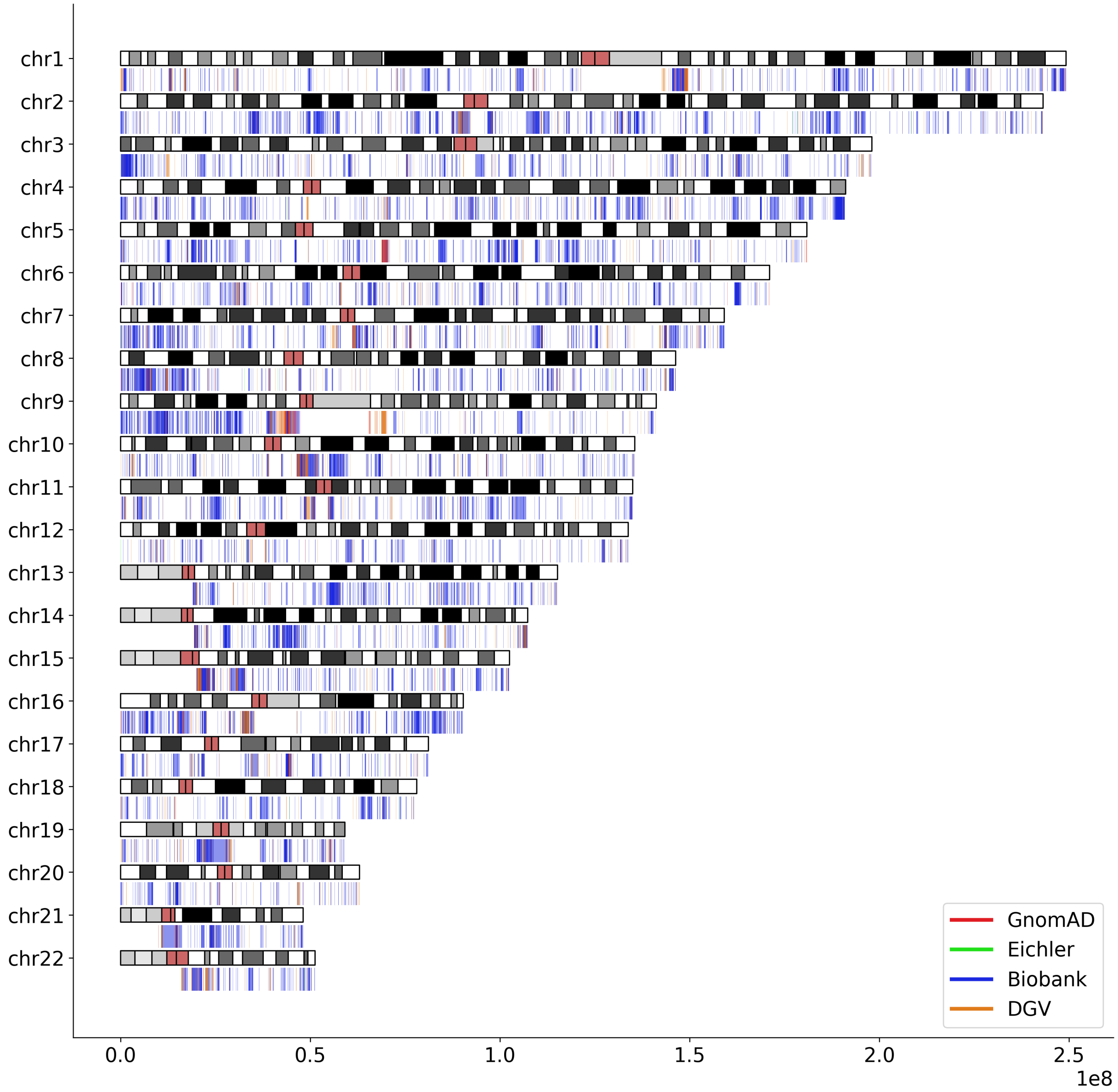

Figure S20. Distribution of Non-Pathogenic Variants across the Genome

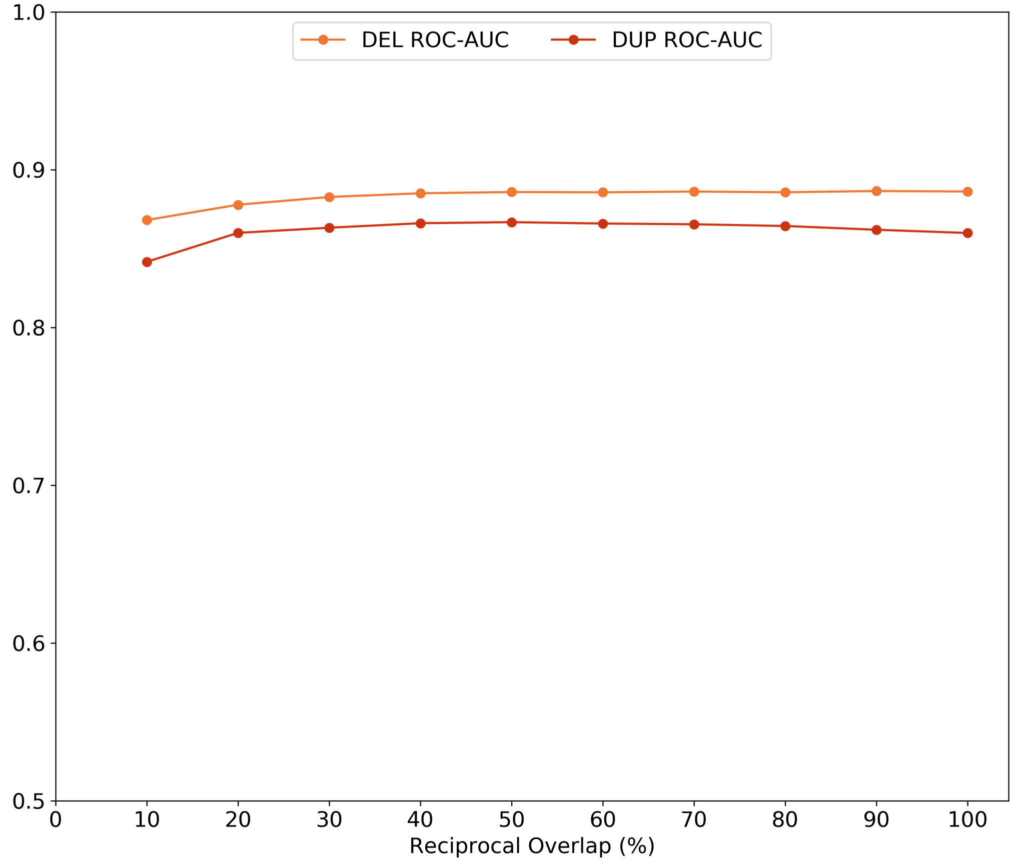

Figure S21. Classification Performance based on Reciprocal Overlap. The figure shows the changes to ROC-AUC values for increasing reciprocal overlap thresholds when comparing ClinVar deletions and duplications to our training data.

Feature	Description
Number of affected Genes	Total number of gene annotations overlapping with the corresponding CNV
Number of affected Enhancers	Total number of enhancer annotations overlapping with the corresponding CNV
Boundary Distance	Distance to the closest TAD boundary (0 if there is an overlap)
Boundary Stability	The stabilitiy i.e. the conservation of the closest TAD boundary across cell types
Gene Distance	Distance to the closest gene (0 if there is an overlap)
Enhancer Distance	Distance to the closest enhancer (0 if there is an overlap)
DDG2P Distance	Distance to the closest gene associated with developmental disease (0 if there is an overlap)
Gene LOEUF	pLoF intolerance of the closest gene
Enhancer Conservation	Primary sequence conservation of the closest enhancer
Gene HI	Predcited Haploinsufficency of the closest gene
CTCF Distance	Distance to the closest CTCF binding site (0 if there is an overlap)
HI Log-Odds-Score	Aggregated predicted haploinsufficiency across all genes overlapping with the CNV
Exon Overlap	Maximum proportion of exons overlapping with the CNV
MPOI	Maximum overlap of a CNV with putative interacting fragments, of each gene in the same TAD environment, normalized by the corresponding pLoF metric.

Table S1. Functional Annotation Based Features for the Training of Pathogenicity Predicting Classifiers.

	**DUP Test Set**	**DEL Test Set**	**ClinVar DEL**	**ClinVar DUP**	**ClinVar DEL (<1Mb)**	**ClinVar DUP (<1Mb)**
**TADA**	0.73	**0.74**	**0.73**	0.53	**0.69**	0.42
**SVScore**	0.43	0.46	0.67	**0.83**	0.66	**0.54**
**VEP**	0.47	0.42	0.69	0.59	0.63	0.43

Table S2. Classification performance by TADA, SVScore, and VEP measured in macro averaged F1 scores on deletions and duplications of the test split as well as ClinVar variants. F1-scores in bold indicate the best-performing method for the individual variant set.

**Variants / Annotations**	**Source**	**Date**
DECIPHER	https://decipher.sanger.ac.uk/	04/25/2019
GnomAD-SV	https://storage.googleapis.com/gnomad-public/papers/2019-sv/gnomad_v2.1_sv.sites.vcf.gz	03/14/2019
Audano et al.	http://ftp.1000genomes.ebi.ac.uk/vol1/ftp/data_collections/hgsv_sv_discovery/working/20181025_EEE_SV-Pop_1/	03/13/2019
UK Biobank	provided by James Priest and Matthew Aguirre (Aguirre et al., 2019)	06/18/2019
DGV	http://dgv.tcag.ca/dgv/docs/DGV.GS.March2016.50percent.GainLossSep.Final.hg19.gff3	01/14/2020
TAD Boundaries + Boundary Stability	https://github.com/emcarthur/TAD-stability-heritability/blob/master/40kbBoundaries/40kbBoundaries_byCellType/H1_ESC_Dixon2015.bed	06/02/2020
CTCF Binding Sites	https://www.encodeproject.org/files/ENCFF453XKM/@@download/ENCFF453XKM.bed.gz	11/27/2019
FANTOM5 Enhancer	https://fantom.gsc.riken.jp/5/datafiles/latest/extra/Enhancers/human_permissive_enhancers_phase_1_and_2.bed.gz	03/19/2019
DDG2P Genes	http://www.ebi.ac.uk/gene2phenotype/downloads/DDG2P.csv.gz	05/07/2019
Haploinsufficiency Predictions	https://decipher.sanger.ac.uk/files/downloads/HI_Predictions_Version3.bed.gz	06/10/2019
pLoF Metrics	https://storage.googleapis.com/gnomad-public/release/2.1.1/constraint/gnomad.v2.1.1.lof_metrics.by_gene.txt.bgz	05/08/2019
pcHi-C	https://www.ncbi.nlm.nih.gov/geo/query/acc.cgi?acc=GSE86189	11/22/2019
HAVANNAH exons	https://www.gencodegenes.org/human/	11/27/2019
Telomeres	https://genome.ucsc.edu/cgi-bin/hgTables	04/18/2019

Table S3. Sources and date obtained for variants and annotation data.
